# Surrogate Biomarkers in Gene Therapy for Orphan Diseases: Validation, Application, and Regulatory Aspects

**DOI:** 10.3390/ijms262010107

**Published:** 2025-10-17

**Authors:** Aisylu I. Ayupova, Valeriya V. Solovyeva, Shaza S. Issa, Haidar J. Fayoud, Albert A. Rizvanov

**Affiliations:** 1 Institute of Fundamental Medicine and Biology, Kazan Federal University, Kazan 420008, Russia; ayimullagulova@kpfu.ru (A.I.A.); vavsoloveva@kpfu.ru (V.V.S.); 2 Department of Genetics and Biotechnology, St. Petersburg State University, St. Petersburg 199034, Russia; shaza.issa98@outlook.com (S.S.I.); haidar.fayoud@gmail.com (H.J.F.); 3 Division of Medical and Biological Sciences, Academy of Sciences of the Republic of Tatarstan, Kazan 420111, Russia

**Keywords:** surrogate endpoints, biomarkers, hereditary diseases

## Abstract

The development of gene therapies for rare hereditary disorders is hindered by small patient cohorts, incomplete characterization of natural disease history, and the impracticality of conducting long-term clinical trials. Surrogate biomarkers—quantifiable indicators predictive of clinical outcomes—represent a promising strategy to accelerate the evaluation of therapeutic efficacy. This review examines the role of surrogate endpoints in gene therapy, outlining essential validation criteria, including biological plausibility, analytical reproducibility, and clinical predictive value. Regulatory frameworks governing surrogate markers in the United States, European Union, Russia, Japan, China, and Canada are compared, with emphasis on mechanisms for expedited or conditional approval. Challenges associated with biomarker validation and extrapolation in the context of rare diseases are discussed, alongside future perspectives that integrate multi-omics technologies and artificial intelligence to enhance biomarker discovery and facilitate regulatory acceptance.

## 1. Introduction

The development of gene therapy in recent decades has marked a new era in the treatment of orphan (rare) hereditary diseases. For many pathologies that were previously considered untreatable, genetic medicines provide fundamentally new opportunities for etiotropic therapy, capable of targeting the root cause of disease—the genetic defect. With rapid advances in molecular biology, viral vectors, and genome editing, the number of clinical trials in gene therapy is increasing each year, while approved products are gradually being integrated into clinical practice [1].

The relevance of this topic lies in the fact that most orphan diseases have a severe progressive course, a limited life expectancy, and are practically unresponsive to traditional treatment methods. Under these conditions, gene therapy becomes not only a high-tech but often the only potentially effective treatment option. However, the development, testing, and registration of such therapies are accompanied by a number of challenges. For instance, clinical studies are hampered by the small number of available patients, considering the prevalence of most orphan diseases is less than 1 case per 10,000 population. In addition, the natural history of many such diseases is only partially understood, and standard clinical endpoints (e.g., survival or functional independence) are often unavailable due to limited observation periods and ethical barriers. Furthermore, these therapies are highly specific and are frequently administered as a one-time treatment, which rules out the possibility of traditional dose optimization and necessitates a fundamentally different approach to evaluating efficacy and safety [2].

Within this framework, surrogate biomarkers—serving as prognostic or pharmacodynamic indicators of treatment response—are critically important. Their application shortens clinical study timelines and enables more objective efficacy assessments within small patient cohorts. Despite their established use in international accelerated approval pathways (e.g., by the U.S. Food and Drug Administration; FDA, and the European Medi-cines Agency; EMA), these approaches remain inadequately formalized within Russia’s domestic regulatory and methodological framework [3].

This review article aims to systematize contemporary perspectives on the application of surrogate biomarkers in gene therapy for orphan diseases. We analyze prevailing strategies for biomarker validation and evaluate the prospects for their integration into clinical development and regulatory assessment. Central to this discussion is an examination of whether these biomarkers can significantly reduce the time required to evaluate therapeutic efficacy. Furthermore, the article provides a comparative analysis of the differing regulatory requirements for surrogate endpoint validation across international jurisdictions.

## 2. Surrogate Markers and Their Classification

### 2.1. Definition of a Surrogate Endpoint (Biomarker)

**Figure 1:**
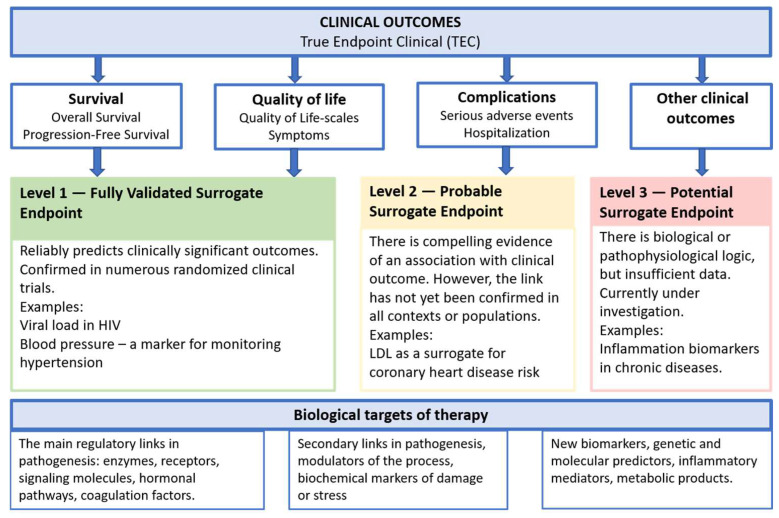
Conceptual relationship between biological targets, surrogate endpoints, and true clinical outcomes.

A surrogate endpoint is a biological marker, laboratory measurement, or clinical parameter used in clinical trials as a substitute for direct, clinically meaningful outcomes [4]. Unlike direct endpoints—such as survival, complication rates, or quality of life—surrogate markers allow for a faster assessment of therapeutic efficacy, thereby reducing the duration and cost of studies [5] (Figure 1).

The pathway from biomarker discovery to regulatory acceptance involves two distinct but interconnected validation processes: biological and regulatory. Biological validation establishes the fundamental “why,” focusing on the marker’s pathophysiological role, its correlation with clinical outcomes in observational studies, and the robustness of its analytical assay. In contrast, regulatory validation demands interventional evidence from clinical trials to prove the “how sure,” requiring that the biomarker not only correlates with the true clinical endpoint but that the treatment’s effect on the clinical outcome is fully captured by its effect on the biomarker [6]. This shift from demonstrating association to establishing predictive causality represents the key conceptual and methodological hurdle, moving from laboratory and epidemiological data to the totality of evidence from controlled trials required for formal regulatory qualification [7]. For a biomarker to qualify as a valid surrogate endpoint, it must meet several key criteria. To begin with, it should correlate with clinical outcomes, meaning that a change in the marker must reliably predict a change in the direct outcome. In addition, a causal relationship must be established, such that an intervention affecting the marker directly influences the clinical outcome. Finally, the biomarker should be relevant to the target population, showing significance for the specific patient group under study [8].

Surrogate endpoints are widely applied in various fields of medicine. In cardiology, lowering low-density lipoprotein cholesterol (LDL-C) is used as a substitute for reducing myocardial infarction risk [9]. In oncology, tumor shrinkage (response measured by RECIST criteria; Response Evaluation Criteria in Solid Tumors) is used instead of overall survival (OS) [10]. Similarly, in endocrinology, normalization of glycated hemoglobin (HbA1c) levels is used instead of directly measuring the incidence of diabetic complications [11].

However, the use of surrogate markers does not always guarantee a reliable assessment of clinical benefit. There have been documented cases where improvement in a biomarker failed to translate into a clinically significant effect. For example, raising high-density lipoprotein cholesterol (HDL-C), traditionally considered “good” cholesterol, did not consistently lower cardiovascular risk. Multiple clinical studies have shown that artificially elevating HDL-C does not necessarily reduce cardiovascular events [12]. This highlights the complexity of the relationship between biomarkers and clinical outcomes. Accordingly, regulatory agencies such as the FDA and EMA require additional validation before accepting surrogate endpoints [12].

While surrogate endpoints play a significant role in accelerating therapy evaluation, their application requires thorough validation. The optimal strategy combines surrogate markers with long-term clinical outcomes, minimizing the risk of misleading conclusions.

### 2.2. Types of Biomarkers

Biomarkers serve as essential tools in modern medicine for diagnosis, prognosis, disease monitoring, and assessment of therapeutic efficacy [13]. Depending on their function, biomarkers can be classified into several types [14].

Diagnostic biomarkers are used to detect or confirm the presence of a disease. They are characterized by high sensitivity and specificity and are frequently applied in screening and emergency diagnostics. For example, the expression of serum microRNA-21 is significantly elevated in breast cancer and serves as a diagnostic biomarker for this malignancy [15].

Prognostic biomarkers predict the natural course or outcome of a disease regardless of treatment. They assess disease severity and the likelihood of complications. For example, mutations in the *KRAS* proto-oncogene are prognostic biomarkers in pancreatic cancer, while mutations in the *TP53* gene indicate an aggressive course of the malignant process [16,17].

Predictive biomarkers are objective biological indicators that can predict the efficacy of certain therapeutic approaches. These markers play a key role in personalized medicine, providing an evidence-based choice for the optimal treatment strategies based on a patient’s molecular profile [18]. For example, activating mutations in the *RAS* genes (*KRAS*/*NRAS*) renders a tumor unresponsive to therapy with monoclonal antibodies against the epidermal growth factor receptor (EGFR) (cetuximab, panitumumab), which is effective only in the absence of such mutations (RAS wild-type). Similarly, the V600E mutation in the *BRAF* gene not only correlates with a more aggressive disease phenotype but also necessitates combination therapy, such as BRAF inhibitors (e.g., encorafenib) together with anti-EGFR agents [19].

Pharmacodynamic (response) biomarkers are used to confirm that the body is responding to therapy, often reflecting early biological changes before clinical effects become evident [20]. An example of such biomarker is the reduction in viral load during antiretroviral therapy [21].

Risk (susceptibility) biomarkers identify individuals at increased likelihood of developing a particular disease, even before clinical symptoms appear. They are often applied in screening programs and genetic counseling. For example, mutations in the *BRCA1/2* genes are associated with a high risk of developing breast and ovarian cancer, and the apolipoprotein E (APOE) ε4 allele is associated with the risk of Alzheimer’s disease [22,23].

The choice of biomarker depends on the specific goals of the study. In practice, the optimal approach often involves combining different types of biomarkers to enhance the accuracy of diagnosis and prognosis [24]. Understanding the strengths and limitations of each biomarker type is therefore crucial for their effective application in both clinical practice and research.

### 2.3. Criteria for a Quality Surrogate Marker

Surrogate endpoints hold great potential to accelerate clinical research by enabling the evaluation of therapy based on intermediate markers rather than waiting for long-term outcomes. However, their application requires a strict evaluation of quality. For a biomarker to be recognized as a valid surrogate endpoint, it must meet a number of key criteria: biological plausibility, reproducibility, predictive value, and regulatory acceptance.

Biological plausibility requires a clear pathophysiological link between the surrogate marker and the clinical outcome. The marker should be directly involved in the disease process or therapeutic response. For example, LDL-C level is involved in atherogenesis, so its reduction is considered a biologically plausible measure for assessing cardiovascular risk. Without such a link, surrogate markers may produce misleading conclusions, as seen with raising HDL-C levels which did not yield the expected reduction in myocardial infarction risk [12].

Reproducibility means that biomarker measurements must be stable, reliable, and consistent across different settings, laboratories, and populations. This is essential for standardization of trial results and obtaining credible data. For example, the HbA1c level is a reproducible and standardized indicator of glycemic control, which has made it an accepted surrogate in evaluating diabetes therapy [11].

Predictive value refers to the ability of a surrogate marker to reliably predict clinically significant outcomes. The marker must show a strong correlation with direct outcomes, and the changes in its level due to therapy should correspond to proportional changes in risk. Predictive value is confirmed statistically through meta-analyses, population studies, and clinical trial data. For example, reductions in viral load in human immunodeficiency virus (HIV) infection are a reliable predictor of slower disease progression and reduced risk of developing acquired immunodeficiency syndrome (AIDS) [21].

Regulatory acceptance by organizations such as the FDA or EMA represents the highest level of validation for a surrogate biomarker. Regulators require not only evidence of correlation and causality but also reproducibility and proven patient benefit. Once recognized, surrogate markers can support accelerated approval of drugs through fast-track access programs or conditional registration [5]. For example, HbA1c is FDA-validated as a surrogate marker in antidiabetic drug trials, while tumor shrinkage according to RECIST criteria is accepted in oncology studies as a temporary substitute for OS [10].

Therefore, a surrogate marker must be more than an easily measurable parameter: it must have strong biological and clinical relevance. Only when all these criteria are met can it be reliably used for making regulatory and therapeutic decisions. Neglecting these requirements increases the risk of misinterpreting results and applying ineffective or even harmful interventions.

## 3. Regulatory Framework and Requirements for Surrogate Biomarkers

### 3.1. Requirements for Surrogate Markers in the United States

The Accelerated Approval program was intended to facilitate earlier access to therapies for serious or life-threatening conditions when no satisfactory treatment options exist. In the program, drug approval may be granted on the basis of surrogate endpoints that are “reasonably likely to predict clinical benefit”. Such markers are based on biological or epidemiological rationale but require further confirmation in post-marketing studies. In contrast, fully validated surrogate markers have undergone extensive evaluation and are known to reliably predict clinical benefit. According to the FDA, 45% of all new drugs approved between 2010 and 2012 were based on validated surrogates [25]. A recent example is Leqembi (lecanemab), approved in 2023 for Alzheimer’s disease under accelerated approval on the basis of reducing brain beta-amyloid levels—a marker considered “reasonably likely” to predict clinical effect [26]. Full, traditional approval followed once confirmatory post-marketing data became available.

The FDA creates and maintains programs for the formal qualification of biomarkers (under the Drug Development Tools (DDT) initiative, specifically the Biomarker Qualification Program). This program allows sponsors to apply for formal qualification of a biomarker for a specific context of use in drug development [27]. Once qualified, the biomarker may be employed across studies without repeated verification. The FDA’s Guidance for Industry documents and the Surrogate Endpoint Resources on its website provide guidance on the use of biomarkers and surrogate endpoints in preclinical and clinical studies [25].

### 3.2. Requirements for Surrogate Markers in Europe

The EMA may grant a Conditional Marketing Authorisation (CMA) for medicines if a series of conditions are met: a positive risk-benefit balance, an urgent medical need, and a commitment to collect confirmatory data post-approval. The conditional authorization is issued for one year and is renewed annually until the data collection confirms the therapy’s sustained effect. Typically, CMA is considered for drugs targeting severe or rare diseases, where clinical datasets are incomplete but potential benefits outweigh risks. The EMA publishes specific obligations for post-marketing studies for each drug. For example, a drug may receive a CMA based on a demonstrated effect on surrogate measures (e.g., biochemical or genetic markers), provided these are later confirmed to translate into an effect on clinical outcomes [28].

The EMA issues specific guidelines regulating the use of biomarkers and endpoints. Key documents include the Guideline on quality, non-clinical and clinical aspects of gene therapy medicinal products (requirements for GTMPs), and the Guideline on clinical trials in small populations (CHMP/EWP/83561/2005), which focuses on special aspects of trials in rare diseases [29]. These and other guidelines (e.g., on adjuvant therapy, bioequivalence, etc.) are considered during the development of experimental drugs. In particular, the EMA notes that studies with small cohorts and surrogate endpoints often require adaptive and flexible statistical approaches, a principle reflected in its regulatory recommendations.

### 3.3. The Use of Surrogate Endpoints as a Tool for Accelerated Drug Registration in the Russian Federation

The challenge of ensuring accelerated patient access to innovative medicines remains one of the key issues in modern clinical and regulatory practice. The Federal Law No. 61-FZ of 12 April 2010, “On the Circulation of Medicines” [30], provides a number of regulatory opportunities to optimize the timelines for state drug registration, particularly through the use of surrogate endpoints, even though it lacks fully formalized procedures equivalent to FDA’s Accelerated Approval or EMA’s CMA.

The Russian legislation permits the use of data from clinical trials conducted outside the Russian Federation, provided they comply with international Good Clinical Practice (GCP) standards article 4, 18 [30]. This allows for the use of international studies based on surrogate endpoints when submitting a registration dossier. Furthermore, as part of integration within the Eurasian Economic Union (EAEU), a system of mutual recognition of registrations has been implemented, which also facilitates an accelerated procedure [31] (Decision of the Council of the Eurasian Economic Commission (EEC) dated 3 November 2016 No. 78 “On approval of the Rules for registration and examination of medicines for medical use” (as amended on 30 January 2020)).

A number of publications emphasize that under conditions of limited time and high medical need, the use of surrogate endpoints can be not only acceptable but a preferable option for assessing efficacy and safety [32,33]. In particular, for the treatment of oncological diseases, the Ministry of Health has already accepted surrogate endpoints such as progression-free survival (PFS) and objective response rate (ORR) as justifications for temporary market authorization.

Another important tool for accelerating registration is the submission of pharmacoeconomic models based on results from surrogate endpoints. This enables the early inclusion of a drug in state funding programs and the List of Vital and Essential Drugs. Thereby, the application of surrogate endpoints can become part of an accelerated access strategy, especially when a dossier is submitted comprehensively with international data, a justification of medical need, and the therapy’s socio-economic significance.

It should be emphasized that this approach requires improvements to the regulatory framework regarding the formalization of mechanisms for conditional or accelerated approval, including the legal recognition of surrogate measures as primary endpoints. This aligns with the global trend toward adaptive regulation in the pharmaceutical industry and is highly relevant in the context of the development of personalized medicine. Table 1 provides a comparative analysis of different regulators’ approaches to the use of surrogate endpoints.

### 3.4. Requirements for Surrogate Markers in Japan

Drug regulation in Japan is governed by the Pharmaceuticals and Medical Device Act (PMD Act) along with subordinate acts, guidance documents, and administrative notifications issued by the Ministry of Health, Labour and Welfare (MHLW) and the Pharmaceuticals and Medical Devices Agency (PMDA). Surrogate endpoints are not explicitly listed in the laws themselves, but their use is considered within clinical requirements. For example, the notification “Basic Principles on Global Clinical Trials” discusses the possibility of using surrogate endpoints provided there is a justified link to the drug’s actual effects [34]. For specific areas, specialized guidelines have been developed; for example, the guideline for anticancer drugs (1991, revised in 2006) initially allowed drug approval based on Phase II trials with tumor response rate as an endpoint, but the 2006 revision requires Phase III data with OS for major cancer types [35]. Thereby, the Japanese regulation relies on international ICH (The International Council for Harmonisation of Technical Requirements for Pharmaceuticals for Human Use) standards (specifically E9/E10) and its own administrative documents, permitting surrogate markers in the presence of convincing evidence of their predictive value [34].

The PMDA, together with the MHLW, is responsible for the scientific review and approval of new drugs. All marketing applications are submitted to the PMDA, which conducts an independent expert assessment of the data and prepares a conclusion for the MHLW [36]. When reviewing an application, the PMDA thoroughly analyzes the choice of clinical trial endpoints. If surrogate endpoints are used in the application, reviewers assess their justification and correlation with clinical benefit. For example, the PMDA’s guideline on global clinical trials notes that surrogates may be acceptable when they reduce sample size requirements, provided there is a reasonable link to a true endpoint (e.g., survival). Protocols must also include secondary endpoints including true outcomes, as well as comparative data from Japanese and global cohorts [34].

The PMDA offers advisory procedures for endpoint selection and grants conditional approval based on stringent criteria. These include the targeting of severe diseases with unmet medical needs, compelling evidence from early-phase studies, and the practical difficulty of conducting full confirmatory trials. Such conditional approval mandates rigorous post-marketing verification of efficacy [36]. Consequently, the PMDA’s role extends beyond document review to actively demanding robust justification for the chosen surrogate endpoint and ensuring a concrete plan for subsequent confirmation of clinical effectiveness. This aligns with the ICH E9 statistical principles, which stipulate that a surrogate’s reliability depends on its biological plausibility, established predictive value for the clinical outcome, and evidence from clinical data demonstrating that a treatment’s effect on the surrogate corresponds to its effect on the final endpoint [37]. To meet this high bar, sponsors must often provide supporting evidence from meta-analyses or historical datasets that validate the relationship between the surrogate and the true clinical outcome.

The PMDA guidelines (including those for medical devices) explicitly require that surrogate endpoints be justified in terms of their relationship to true effects [34] (Release of Clinical Trial Guidance to Facilitate the Speedy and Accurate Approval and Development of Medical Devices). The PMDA adopts a logic similar to the FDA’s classification of surrogates into “validated” versus “reasonably likely predictors”. If the surrogate is not fully validated, PMDA typically mandates confirmatory studies or long-term re-examination (usually after 6–8 years) [38].

When submitting a drug registration application, a company provides full clinical study reports justifying the choice of primary and secondary endpoints. If a surrogate is claimed, the PMDA usually requires the inclusion of both “hard” primary and secondary outcome measures for subsequent analysis [34].

Upon completion of the review and upon granting marketing authorization, a re-examination period may be assigned, during which companies are obligated to collect additional clinical data. In oncology, for example, confirmatory OS trials following surrogate-based approval have been completed for only ~17–18% of drugs, and in some cases, confirmation has been delayed or cancelled [39]. This highlights the need for a strict process: the PMDA may require a confirmatory Phase III trial with a “hard” endpoint or mandate additional data collection, especially if the drug’s effect is questionable. The influence of international trials is also considered: the PMDA encourages multinational studies with a unified methodology, but the applicability of global data to Japanese populations must be justified. In summary, the procedure for considering surrogate markers in Japan combines requirements for justified endpoint selection, confirmatory statistical power, and, if necessary, subsequent post-marketing control.

The PMDA, like the FDA and EMA, accepts these markers provided their role is proven. In general, Japan, following global approaches, often permits the same surrogates for drug approval as the US and EU, while accounting for national trial design specifics.

### 3.5. Requirements for Surrogate Markers in China

In China, the regulation of surrogate markers is defined by national legislation and sector-specific regulatory documents. The key framework is the Drug Administration Law, (revised in 2019) together with the Drug Registration Regulation (July 2020), which formally introduced accelerated approval pathways. In addition, the National Medical Products Administration (NMPA) and its Center for Drug Evaluation (CDE) regularly publish technical guidelines on clinical endpoints for specific diseases. For example, in the guidance on advanced non-small cell lung cancer, the regulator explicitly prioritizes “direct” clinical outcomes (OS, symptom improvement), but also allows surrogate endpoints that are “reasonably likely to predict clinical benefit”, such as a high objective response rate (ORR) with sufficient duration of response (DOR) [40]. Thereby, the Chinese regulatory framework (laws and NMPA orders) formally recognizes the use of surrogates, provided there is a scientific justification.

For drugs seeking accelerated or conditional approval, the NMPA has established special procedures. Under the Technical Guideline on Conditional Approval, if a disease is life-threatening and there are no effective alternatives, a drug may be conditionally approved based on a surrogate endpoint or an “intermediate” clinical endpoint [41]. The chosen surrogate must have a plausible biological rationale and a demonstrated statistical association with the expected clinical benefit. Final “full” approval is granted only after confirmatory post-marketing or randomized controlled trials (RCTs) with clinical endpoints (the completion of such trials is mandatory). Overall, the process parallels the FDA’s accelerated approval and the EMA’s conditional marketing authorization, but in China it is formalized by the legislative framework of “Conditional Approval” (introduced following the 2019 amendments and 2020 orders).

The Chinese regulators set strict requirements for surrogate validation. Scientific and statistical criteria include: evidence of a biological link between the marker, disease pathology, and clinical outcome; proven correlation between changes in the marker and patient improvement; and reproducibility of measurement. Guidelines stress the need for “biological plausibility” of the surrogate–outcome relationship and supporting epidemiological or clinical data. In other words, a convincing mechanism, such as a genetic or molecular pathway, must demonstrate how marker changes translate into clinical benefit. Chinese experts also emphasize the importance of clinical validation data: it must be shown that an effect on the surrogate corresponds to a real clinical effect.

The CDE has approved a technological standard whereby, in pilot trials for postmenopausal osteoporosis, the change in bone mineral density (BMD) is often accepted as a surrogate marker. In the “Pilot” study phase, percentage change in lumbar spine BMD (L1–L4) is recommended. However, for confirmatory RCTs the primary clinical endpoint is considered to be the risk of new pathological vertebral fractures, while increased BMD is considered supportive but not definitive. This aligns with the international approach: an increase in BMD is associated with a reduction in fractures, but the ultimate goal always remains the reduction in clinical events [42].

A similar principle applies to oncology. For example, the guideline for multiple myeloma states that the level of minimal residual disease (MRD) is “sufficiently closely correlated with the risk of relapse and long-term prognosis”, but “the degree of association may change as treatment outcomes improve”. Similarly, in the guidelines of EMA any novel surrogate endpoints used to assess benefit–risk require “comprehensive confirmation of clinical validity”. Accordingly, the Chinese framework aligns with international standards (ICH, FDA, EMA), while underscoring the need for strict scientific evidence linking surrogate markers to true clinical benefit.

### 3.6. Requirements for Surrogate Markers in Canada

Canada does not have a separate law dedicated to surrogate endpoints; instead, their use is regulated under the general framework for pharmaceuticals. The core legislation is the Food and Drugs Act and its associated Food and Drug Regulations (Division C.08, New Drug Submissions). Health Canada, through its Health Products and Food Branch (HPFB), issues policies and guidance documents (e.g., on accelerated programs and Notice of Compliance with Conditions, NOC/c), that specify evidentiary standards for surrogate use. For instance, the guidance on Section 3/Appendix A of the Food and Drugs Act notes that “surrogate endpoints may be used to assess the risk of a disease provided that the relationship between the surrogate endpoint and the disease has been definitively established” [43]. Furthermore, Canada participates in the ICH; its regulator adheres to ICH recommendations (e.g., ICH E3, E9). Specifically, the ICH E3 guideline (applied in Canada) states that if a surrogate endpoint was used in a clinical trial, its selection must be justified with references to data, publications, or prior regulatory decisions [44].

Health Canada evaluates clinical data for drugs (through the Therapeutic Products Directorate) and determines whether surrogate markers are sufficient to draw conclusions about efficacy. Under the NOC/c policy, surrogate markers are defined as parameters that, “when directly measured, are expected to predict the drug’s effect on recognized clinical outcomes (morbidity, mortality)”. It is emphasized that a validated surrogate “predicts the clinical benefit of the drug”. Non-validated surrogates, however, cannot substitute for clinical outcomes; in such cases, Health Canada requires confirmation through additional studies [45].

When reviewing registration dossiers (NDS/SNDS), Health Canada focuses on the totality of evidence for efficacy. If a surrogate is chosen as an endpoint, the sponsor must provide rationale for its validity (biological link, literature, experience of other regulators) and commit to confirmatory studies. As per ICH E3, surrogate endpoints must be justified with data or regulatory references [44]. Health Canada also recommends early consultation with the relevant department before trial initiation, and also providing appropriate evidence of validity [43]. Ultimately, the regulator evaluates surrogate markers alongside any clinical outcomes and makes a market access decision: either a standard NOC (full approval) or NOC/c (conditional approval with post-market obligations), depending on the degree of data uncertainty.

Canadian practice reflects international experience. For example, viral load suppression in HIV (as it is associated with slowed AIDS progression), and tumor shrinkage (ORR) in oncology are established surrogates [45]. PFS is commonly used as a predictor of OS in cancer trials.

Similarly, antibody titers after vaccination serve as predictors of immunization and protection against disease [45]. Although the direct clinical outcome is infection, antibody levels often serve as a proxy measure of efficacy.

In summary, Canada, the U.S., and the EU follow similar principles of accelerated access based on surrogate endpoints, although with some differences.

## 4. Preclinical Studies and Translational Biomarkers

### 4.1. Transgenic and Knockout Animals for Modeling Rare Diseases

In preclinical gene therapy research, transgenic and knockout animal models are widely used to relevantly reproduce the pathogenesis of specific orphan diseases. These models allow evaluation of the correction of pathological parameters (e.g., reduction in toxic metabolite accumulation or restoration of enzymatic activity) serving as valid proof-of-concept evidence of therapeutic efficacy. For example, It has been established that in a mouse model of mucopolysaccharidosis type I (MPS I) (α-L-iduronidase (IDUA) deficiency, IDUA^−^/^−^ line), gene therapy normalizes levels of heparan and dermatan sulfates in tissues and urine, and reduces the HCII–thrombin complex, a biomarker of disease severity [46,47].

The critical role of surrogate biomarkers is evident in preclinical models that re-capitulate biochemical pathology but not the full clinical spectrum of the disease. In the ARSA^−^/^−^ mouse model of metachromatic leukodystrophy (MLD), treatment with AAV-ARSA gene therapy significantly elevates ARSA enzymatic activity in the brain and reduces systemic sulfatide accumulation [48]. However, because this model is considered “non-diagnostic” due to its minimal demyelination, the assessment of therapeutic efficacy relies entirely on these biochemical markers rather than on motor function [49]. A related model ASA^−^/^−^ + hASA-C69S, similarly lacks full demyelination [50].

The same principle applies to Tay-Sachs disease (TSD) research; HEXA^−^/^−^ mice exhibit the characteristic reduction in β-hexosaminidase activity and accumulation of GM2 ganglioside but display almost no neurological symptoms [51]. Consequently, the level of GM2 or its plasma derivative, Lyso-GM2, serves as the essential biomarker for evaluating treatment candidates [52]. These examples underscore that surrogate endpoints are vital for providing proof-of-concept in models where a clear phenotypic readout is absent.

For hemophilia B, the standard model is mice with a knockout of the F9 gene (F9^−^/^−^ line), which demonstrate a pathologically reduced plasma activity of coagulation factor IX (FIX) (<1% of normal). Administration of a therapeutic *FIX* gene using an adenoviral vector leads to a dose-dependent restoration of coagulant activity to physiological levels [53].

Gaucher disease, a lysosomal storage disorder (LSD) caused by β-glucocerebrosidase (GBA1) deficiency, is modeled in mice with a complete knockout GBA1^−^/^−^ or mutations in this gene. Such mice demonstrate characteristic accumulation of glucocerebroside (GlcCer) and glucosylsphingosine (lyso-Gb1) in macrophages and target organs (liver, spleen, bone marrow, and also the CNS in neuronopathic forms) [54,55]. Gene therapy with viral vectors (AAV, lentiviral) in GBA1-deficient mice restores gene expression and GBA1 activity, reduces lipid accumulation, and improves histological and clinical parameters [56,57]. The effectiveness of therapy is monitored by the level of lyso-Gb1, which is recognized as the most sensitive and specific biomarker of the disease [58].

Fabry disease results from α-galactosidase A (GLA) deficiency, causing accumulation of globotriaosylceramide (Gb3) and its toxic derivative lyso-Gb3 in the kidneys, heart, skin, vessels, and CNS [59]. GLA^−^/^−^ mice were created to replicate this pathology, exhibiting substrate accumulation similar to humans [60]. Such model animals allow for the assessment of pathological sign correction: a reduction in Gb3 and lyso-Gb3 levels in tissues and plasma, restoration of enzymatic activity, and improvement of organ histological characteristics. These changes serve as proof-of-concept for the efficacy of gene therapy. For example, in AAV-mediated delivery of a functional *GLA* gene to model mice, a reduction in Gb3, a sensitive biomarker of disease severity, is observed [61,62].

Ethical and methodological challenges also arise in the selection of appropriate translational models. Although useful, small animal models like mice often fail to fully recapitulate human disease phenotypes due to interspecies differences in metabolism, immunity, and neurological complexity. These limitations underscore the need for large animal models (e.g., dogs, sheep, nonhuman primates) that better mimic human anatomy, physiology, and biodistribution. Consequently, large models are invaluable for assessing the safety, immunogenicity, and biodistribution of gene therapies [63,64].

### 4.2. Correlation with Clinical Pathology

For successful translation of preclinical findings into clinical application, bridging biomarkers are required—universal quantitative indicators measurable both in animal models and in patients. Such biomarkers should reflect key pathophysiological mechanisms and enable prediction of therapeutic efficacy in humans based on preclinical data [65]. An ideal surrogate should correlate closely with the clinical picture: for example, a reduction in glycosaminoglycans (GAG) in mice should be accompanied by a similar reduction in GAG in MPS I patients [66]. Plasma levels of lyso-Gb1 are high in Gaucher disease and decrease sharply with therapy, demonstrating high sensitivity in monitoring patient status [67]. In MLD, a reduction in sulfatide accumulation reflects similar changes in patients. Furthermore, potential interspecies differences (e.g., incomplete demyelination in ARSA^−^/^−^ mice) must be considered, requiring careful interpretation when extrapolating data to humans [68]. The level of N-acetylaspartate (NAA) in the brain in Canavan disease is a marker of neuronal integrity; its increase after therapy signals an improvement in neuronal function [69].

Consistency of responses across species confirms the relevance of the model. Reliable analytical methods are essential: the sensitivity and specificity of tests for enzymes or metabolites must be validated according to FDA/EMA standards.

### 4.3. Regulatory Requirements for Preclinical Biomarker-Based Studies

Regulators impose strict standards on biomarker studies at the preclinical stage. To Regulators impose strict standards on biomarker studies at the preclinical stage. To begin with, the analytical method must be thoroughly verified: enzyme or metabolite tests must have known sensitivity, specificity, reproducibility, and stability. Moreover, the experimental design must ensure sufficient statistical power: the number of animals and replicates is determined in advance, taking into account the expected therapy effect. Independent replication of results and control of all potential sources of variability are also important [70]. Overall, biomarkers require extensive method validation (as in bioanalytical studies) and regulatory-mandated rigor in data handling (Good Laboratory Practice (GLP) principles, etc.). Failure to meet these standards often results in poor reproducibility of results and reduced confidence in surrogate endpoints [65].

## 5. Surrogate Biomarkers and Clinical Studies of Gene Therapy Products

### 5.1. Selection and Justification of a Surrogate Marker in Phase I–III Protocols

When developing clinical protocols, it is necessary to justify the choice of a surrogate marker: it must have a clear biological rationale, adequately reflect the pathophysiology of the disease, and correlate with the clinical outcome [25]. For biological relevance, the marker should be closely linked to the mechanism of action of the therapy (e.g., ARSA levels in MLD, FIX levels in hemophilia B). The evidence base includes retrospective data or natural history information associating the marker with disease severity or progression. For instance, it has been demonstrated that low HexA activity in TSD patients is accompanied by elevated Lyso-GM2 levels [52]. Data from prior studies or correlations must indicate that changes in the marker lead to changes in clinical status. Additionally, the methodology used for measuring the marker in clinical settings must be reliable and reproducible.

Integration of natural history studies with biomarker research is a crucial component of surrogate validation, particularly in rare diseases. Natural history datasets help identify measurable parameters that correlate with disease progression and can later serve as surrogate endpoints in therapeutic trials. By linking biomarker changes to longitudinal clinical outcomes, such studies provide an evidentiary bridge between biological relevance and regulatory acceptance.

Regulators recommend developing these justifications in dialogue with experts and relying on existing guidance. For example, the FDA notes that acceptance of a surrogate requires a “substantial body of evidence” (epidemiological and clinical), demonstrating the marker’s association with clinical benefit [25]. In practice, this means that Phase I-III protocols must detail the logic behind the use of the marker and, where possible, provide a retrospective analysis of historical data.

### 5.2. The Role of Biomarkers in Accelerated/Conditional Approval

In the context of severe rare diseases, regulators may accept “promising” surrogates as endpoints in accelerated approval pathways. The FDA allows surrogate endpoints that are “reasonably likely to predict clinical benefit” to be used for accelerated approval, particularly when conducting full-scale clinical trials is challenging [25]. For example, in hemophilia B programs, the percentage of normal FIX activity is used as a secondary marker; its increase is considered a reliable indicator of reduced bleeding risk. Therefore, in the approval of the gene therapy drug Hemgenix (etranacogene dezaparvovec) the primary endpoint was FIX levels (along with bleeding history) [53,71]. Similarly, in LSDs, specific substrate levels (e.g., urinary or cerebrospinal fluid (CSF) GAGs) are planned as endpoints. Accordingly, under conditional or accelerated approval, metabolic biomarkers are often accepted as primary endpoints, with the expectation that their predictive validity will be confirmed in subsequent studies.

### 5.3. Post-Marketing Studies

After a gene therapy product receives marketing authorization, continued monitoring of its clinical efficacy and safety is required. Regulators typically mandate confirmatory clinical trials to verify that improvement in the surrogate indeed corresponds to an improvement in clinical outcomes. In addition, long-term follow-up (LTFU) is obligatory for gene therapies, for instance, according to FDA recommendations, patients may be tracked for up to 15 years to identify long-term adverse effects and durability issues [25,71] (FDA-2018-D-2173). Market authorization is maintained only if the surrogate’s expected effect is confirmed. If subsequent data fail to confirm therapeutic benefit, approval may be withdrawn. Such cases have already occurred outside of gene therapy: for example, drugs with accelerated approval in oncology and neurology (Oxbryta for sickle cell disease and Aduhelm for Alzheimer’s disease) were later withdrawn after failure of confirmatory trials [72,73]. These examples show that even under the pressure of disease rarity, regulators insist on thorough post-approval control. To date, no such withdrawals have occurred for approved gene therapies; however, trends indicate the necessity of adhering to the highest evidentiary standards at every stage of testing.

## 6. Key Examples of the Use of Metabolic Surrogate Biomarkers

### 6.1. Metachromatic Leukodystrophy

Metachromatic leukodystrophy (MLD) is an inherited demyelinating disorder caused by a deficiency of ARSA. Insufficient ARSA leads to accumulation of galactosylceramide-3-O-sulfate (sulfatides) in the central (CNS) and peripheral (PNS) nervous system, resulting in progressive demyelination of nerve fibers [49,74]. The degree of sulfatide accumulation closely correlates with the severity of neural tissue damage [48]. The change in sulfatide concentration is used as a metabolic surrogate in MLD therapy: a reduction in their level is considered an indicator of resumed lipid degradation and potential myelin restoration [75]. Preclinical studies confirm that effective gene therapy can completely eliminate sulfatide accumulation (in mice, intracerebrovascular administration of AAV-ARSA resulted in “full normalization of sulfatide accumulation” and improvement of inflammatory response) [76]. Sulfatide isoforms are considered well-established biomarkers of MLD [48].

One of the key therapies for MLD is Libmeldy (atidarsagene autotemcel)—an ex vivo gene therapy based on autologous CD34^+^ hematopoietic stem cells (HSCs) transduced with a lentiviral vector encoding the *ARSA* gene (LV-ARSA) [77]. Libmeldy is approved by the EMA and FDA for treating children with early-onset and juvenile forms of MLD (while still asymptomatic or with early manifestations) [78,79]. In clinical trials (NCT01560182), ARSA cell therapy resulted in significant increases in enzyme activity in blood and preservation of motor and cognitive function at levels comparable to healthy peers [80]. Long-term follow-up demonstrated that most early-juvenile patients maintained normal cognitive and motor development. The primary endpoints in these studies included clinical motor function assessment (Gross Motor Function Measure (GMFM)) and biochemical parameters (ARSA activity, sulfatide levels) [79,81].

In addition to ex vivo gene therapy, direct administrations of vectors carrying the *ARSA* gene are being investigated. In preclinical experiments, recombinant AAV vectors (intravenous or intrathecal administration of AAV-PHP.eB-ARSA) normalized sulfatide levels in the CNS, with reduction in neurological dysfunction and inflammation [76]. In China, a clinical trial of LV-ARSA gene therapy targeting the CNS is ongoing (NCT03725670), although no data have yet been published.

Enzyme replacement therapy (ERT) is also being explored. A phase I/II trial of intrathecal administration of recombinant human ARSA (rhASA) (NCT01510028), showed that after repeated injections in children with MLD, sulfatide and lysosulfatide levels in CSF decreased to normal, while the rate of motor decline slowed. These results support the concept of CSF sulfatide reduction as a surrogate of efficacy: normalization of sulfatide levels in the CSF indicates enzyme delivery and the initiation of breakdown of accumulated lipids [82]. Therefore, in MLD, a reduction in metabolites (sulfatides) in CSF/tissues is considered a valid surrogate for improved myelination and clinical benefit [48,82].

### 6.2. Mucopolysaccharidosis Type I

Mucopolysaccharidosis Type I (MPS I) is an LSD associated with deficiency of IDUA. Lack of IDUA results in accumulation of heparan sulfate and dermatan sulfate across multiple tissues, leading to multi-organ dysfunction (cardiomyopathy, hepatic and splenic insufficiency, neurodegeneration) [83]. In MPS I, accumulated GAG levels, particularly in urine and CSF, are used as biomarkers of therapeutic efficacy. Measurement of urinary GAGs has long been used for diagnosing and monitoring MPS, as a reduction in urinary GAG excretion serves as a direct indicator of substrate clearance [84].

Aldurazyme (laronidase) is an ERT using recombinant human IDUA. In clinical trials and practice, it shows significant GAG reduction. In a randomized study, 26 weeks of laronidase therapy led to ~54% reduction in urinary GAG excretion, whereas placebo patients showed a 47% increase [85]. This confirms the role of GAG levels as a reliable surrogate: their reduction correlates with stabilization or improvement of organ manifestations of MPS I. Aldurazyme has been approved by FDA and EMA for long-term ERT in MPS I since 2003.

The phase III OTL-203 trial (NCT06149403) evaluates ex vivo gene-cell therapy using lentivirally modified HSCs encoding *IDUA*. In 2023, the FDA approved the investigational new drug application [86]. Secondary endpoints include biochemical markers (such as urinary/CSF GAGs, etc.).

Both FDA and EMA recognize normalization of urinary/CSF GAGs as a valid biochemical surrogate of clinical benefit in MPS I [84,85]. For example, reductions in dermatan sulfate and heparan sulfate are considered plausible evidence of clinical effect in long-term therapy. However, regulators also require demonstration of actual clinical benefits (functional improvement, morbidity reduction). In new randomized studies (including OTL-203), outcomes such as neurodevelopment, brain morphology, etc. are assessed alongside surrogates. Nevertheless, reduced GAGs have already formed the basis of most approved drugs for MPS I and many design criteria for current gene therapy protocols.

### 6.3. Tay–Sachs Disease

Tay–Sachs disease (TSD) is a hereditary GM2-gangliosidosis caused by a defect in the α-subunit (*HEXA* gene) of β-hexosaminidase A (HexA). Deficiency of HexA prevents GM2 degradation, leading to its pathological accumulation in neurons and glial cells, causing neurodegeneration and early death [51,87]. Currently, only supportive treatments are available for TSD, but gene therapy is under intensive development. Studies on animal models (murine, ovine, feline) show that GM2 tissue/CSF levels correlate with disease severity, while HexA activity indicates metabolic status. Therefore, experimental interventions primarily focus on these biomarkers.

Genetic models of GM2-gangliosidoses include *HEXA* or *HEXB* knockout mice, Korat cats, Sandhoff cats (SD, mutations in the gene encoding the β-subunit of HexA: HEXB) [88] and Jacob sheep with true TSD [51]. Considering that mice have alternative ganglioside metabolism (attenuating TSD phenotype), Sandhoff cats and Jacob sheep are often used as more clinically relevant models [89]. In these models, gene delivery methods, assessment of GM2 levels (in the brain, CSF), and HexA activity are tested as efficacy criteria.

For TSD, the main surrogates are GM2 accumulation and HexA levels. Elevated GM2 can be measured biochemically in tissues and CSF; after successful therapy, it should decrease. In parallel, HexA activity (in blood/CSF) should increase, reflecting function of the delivered gene.

Gene therapy with AAVrh8-HEXA and AAVrh8-HEXB in two infants with infantile TSD showed an increase in HexA activity, compared to baseline values in the CSF. In one patient, transient stabilization and continued myelination were observed on magnetic resonance imaging (MRI), temporarily “deviating” from the expected natural course [90]. This demonstrates that AAV-based therapy can deliver enzyme to the brain, with HexA activity and GM2 levels in CSF serving as key surrogates. The results have not yet given definitive clinical improvement, but the approach has opened the way for further studies.

To date, there is no formally recognized surrogate for TSD. Given its severity and rarity, significant improvements in GM2/HexA are accepted as evidence of biological effect, to be further confirmed by clinical outcomes (neurological scales, neuropsychological testing). Additional measurements of myelin-related biomarkers (e.g., diffusion on MRI) and inflammatory cytokines are planned as potential surrogates of metabolic correction.

### 6.4. Hemophilia B

Hemophilia B is an inherited deficiency of FIX, resulting in impaired coagulation and risk of spontaneous or traumatic bleeding. In patients with low FIX activity (<1%), even minor injuries cause severe hemorrhages into joints, muscles, and vital organs [91]. In gene therapy for hemophilia B, the surrogate endpoint is considered to be increased FIX activity in the blood, since higher activity of this indicator correlates with reduced bleeding frequency. For example, in trials of Hemgenix (etranacogene dezaparvovec, AAV5-Padua-FIX, approved in 2022) mean *FIX* expression reached 36–41% of normal, accompanied by an approximate two-fold reduction in the annual number of bleeding episodes (annualized bleeding rate, ABR) [92] (Clinical Review Memo, 22 November 2022—HEMGENIX).

In the initial studies (phase III, NCT03569891) the primary endpoint was ABR before and after therapy. The FDA review reported mean ABR reduction from 4.1 to 1.9 bleeds/year, following vector infusion, while FIX activity stabilized at ≈37–39% (Clinical Review Memo, 22 November 2022—HEMGENIX). Based on these data, FDA concluded the gene product was effective, with FIX activity considered a “reasonably likely surrogate” of reduced bleeding. However, regulators required further confirmatory research. Specifically, the FDA included in its post-marketing requirements an analysis of the relationship between anti-AAV5 antibodies, FIX expression levels, and bleeding risk. Thereby, the approval was based on a combination of biochemical (FIX activity) and clinical (ABR) data, with regulatory oversight ensuring that the true reduction in bleeding episodes is confirmed in long-term follow-up.

## 7. Potential Challenges and Limitations

### 7.1. Insufficient Validation of Surrogate Endpoints

The main risk of using surrogate markers is that changes in a biomarker do not necessarily reflect a true clinical effect. As Fleming noted, “correlation does not make a surrogate”, even if a marker is associated with a disease, therapeutic intervention on it may not translate into patient benefit. In a number of well-known cases from different medical fields, changes in surrogates failed to produce effects on “hard” clinical outcomes. For example, in cardiology, antiarrhythmic therapy after myocardial infarction substantially reduced the frequency of ventricular arrhythmias but unexpectedly increased the risk of sudden death (The Cardiac Arrhythmia Suppression Trial, CAST) [93]. Similarly, in postmenopausal women, hormone replacement therapy improved lipid profiles but increased rates of myocardial infarction and mortality [94]. In osteoporosis, fluoridation increased bone density but the number of fractures increased [95]. In ophthalmology as well, lowering intraocular pressure did not protect against visual field loss in glaucoma [96]. Finally, in oncology, the widely used surrogate prostate-specific antigen (PSA) level in prostate cancer did not demonstrate a reliable effect on OS [97].

These examples show that interpretation of clinical trial results based on surrogate markers must be very cautious. Strict statistical evidence is required to prove that changes in a surrogate reliably lead to the expected clinical effect. In many cases, meta-analyses have shown that outcomes such as survival cannot be predicted with confidence from surrogate responses. This highlights the importance of full biomarker validation: without evidence that the surrogate reliably correlates with clinical outcomes, the risk of mistaken decisions is high.

Quantitative analyses illustrate the risk of relying on surrogate endpoints without rigorous validation. A number of accelerated approvals based on surrogate measures have required long intervals for confirmatory trials (median projected delay ~4–5 years in non-oncology approvals), and when confirmatory trials are not completed or fail to show clinical benefit drugs or indications may be withdrawn [98]. These data underline that surrogate-based approvals must be supported by robust analytic and longitudinal evidence (e.g., meta-analytic validation or strong mechanistic causal data) to reduce the risk of post-marketing failure. Furthermore, both FDA and EMA now encourage the use of real-world data (RWD) and real-world evidence (RWE) to complement clinical trial datasets, strengthening post-approval monitoring and validation of surrogate endpoints [99].

### 7.2. Complexity of Extrapolation

Another fundamental challenge is the persistent translational gap between preclinical models and human patients. Biological differences in disease pathogenesis, metabolism, and pharmacokinetics mean that interventions successful in animal models often fail to reproduce their effects in human trials. A prominent example is in neurodegenerative disease, where numerous compounds that effectively reduced β-amyloid in mouse models did not improve cognitive function in patients [100]. This disconnect means that positive effects on a surrogate endpoint in a preclinical setting do not guarantee clinical efficacy. Consequently, the path to regulatory acceptance is fraught with additional hurdles. Regulators require prospective clinical trial evidence proving that a change in the biomarker reliably predicts clinical benefit—a costly and lengthy validation stage where many candidates fail. Further complicating this transition are challenges such as a lack of analytical standardization across clinical sites, in-sufficient statistical power in rare disease cohorts, and a failure to precisely define the biomarker’s context of use, all of which hinder the journey from a mechanistically interesting finding to a regulatorily endorsed tool [101,102,103].

### 7.3. Rare and Ultra-Rare Diseases

In the context of rare diseases, the limitations of surrogates become particularly critical. Small cohorts and the absence of historical data aggravate the problem. With a limited number of patients, it is difficult to gather a statistically significant sample, and the effects of any biomarker may become insignificant due to high variability. As noted in an analysis of clinical trials in rare genetic diseases, “interpretation of results in many studies is complicated by small sample sizes, the absence of internal or historical controls, and limited use of quantitative measurements” [103]. This makes validation of even physiologically plausible markers (e.g., enzyme or metabolite levels) problematic.

Moreover, rare diseases often lack long-term epidemiological data or registries that could verify biomarker–outcome associations. Without retrospective series, it is difficult to reliably demonstrate that a surrogate change corresponds to functional improvement or survival. Consequently, in rare diseases, standards for surrogate validation are typically less strict, indicative or combined endpoints (e.g., biomarker level combined with a clinical test) are often accepted. However, this increases the risk of adopting clinically irrelevant surrogates. Finally, in ultra-rare syndromes with extremely small cohorts, special flexible statistical strategies are applied (such as Bayesian methods or relaxed significance thresholds), but these approaches require additional hypothesis testing and may weaken the accuracy of evidence. Collectively, small sample sizes, lack of historical data, and ethical constraints (e.g., inability to conduct large placebo-controlled trials) make justification of surrogate endpoints in rare diseases one of the most challenging stages of therapy development [103,104].

## 8. Trends and Future Prospects

### 8.1. Novel Technologies for Biomarker Identification and Validation

Rapid technological progress is opening new horizons for biomarker discovery and validation. A particularly significant advance is the transition to multi-scale “omics”: genomics, transcriptomics, proteomics, metabolomics, and others. Integrating various omics data allows for the identification of previously unknown interrelationships and complex pathophysiological signatures [105]. Modern high-throughput methods (mass spectrometry, sequencing, microarrays), combined with machine learning and artificial intelligence (AI), enable the analysis of vast datasets and the selection of promising marker combinations. For example, there are already approaches for automated biomarker candidate selection based on deep neural networks, trained on large clinical and genetic datasets [106,107]. The combined use of “big data” and AI also helps account for gene polymorphisms and evaluate genetic tests in the context of rare diseases. Accordingly, advanced algorithms can accelerate biomarker discovery: in screenings of tens of thousands of MRI scans or proteomic spectra, they can identify signature clusters most relevant to disease [101,105]. The integration of multi-omics data with AI follows a structured pipeline for biomarker discovery. This process begins with the generation and harmonization of high-dimensional data from patient biospecimens, which is then mined using machine learning to identify complex biomarker signatures. AI-prioritized candidates subsequently undergo biological validation in disease models, followed by the development of robust clinical assays. These lead biomarkers are then tested in prospective clinical studies, where refined AI models establish their predictive value for patient outcomes. The accumulated evidence is finally compiled for formal regulatory qualification, creating an iterative pipeline that accelerates the development of accurate, multi-analyte biomarkers [108].

### 8.2. Expansion of Regulatory Support

Regulators actively create frameworks for the recognition of new biomarkers and surrogates. In the United States, the FDA Biomarker Qualification Program aims to collaborate with researchers and companies to formalize biomarkers as drug development tools [109]. The FDA may issue Letters of Support for promising biomarkers, formally acknowledging their importance and encouraging collaborative research. Similar initiatives are emerging in Europe: EMA conducts the “Qualification of Novel Methodologies” procedure, through which CHMP may issue qualification advice or opinions on biomarkers. For particularly promising methodologies, EMA also issues public Letters of Support, promoting data sharing and further validation [110].

Finally, the role of clinical data transmission standards is growing. The Critical Path Institute (C-Path) and Clinical Data Interchange Standards Consortium (CDISC), through the CFAST (Coalition for Accelerating Standards and Therapies) alliance, are developing therapeutic area user guides (TAUGs) and ontologies for various therapeutic areas. CDISC standards for specific diseases (including rare ones) help standardize endpoint, laboratory, and biomarker reporting. Such standardization accelerates data aggregation and processing: adoption of CDISC formats has been shown to “reduce time and resource needs” in clinical studies, while C-Path databases built on these formats provide “high-quality data for disease modeling and analytics”, thereby facilitating biomarker qualification [111]. CFAST was officially launched “to accelerate research and therapy development through establishing and supporting data standards, tools, and methods”. Thanks to these regulatory and standardization initiatives, biomarker qualification and validation are becoming more transparent and predictable.

Rare disease patient organizations and foundations play a pivotal role in biomarker development. Through patient registries, funding of multicenter studies, and data-sharing initiatives, groups such as NORD, EURORDIS, and Global Genes help identify clinically relevant surrogate markers and facilitate their validation. These partnerships between patient communities, academia, and regulators accelerate both discovery and acceptance of surrogate biomarkers [112].

### 8.3. Integrated Approaches

Modern studies increasingly use combined endpoints, integrating biochemical markers with clinical measurements. For example, biomarker dynamics may be analyzed alongside neuropsychological test scores or physical function scales. Such approach enhances the reliability of conclusions as the marker would be confirmed by a “hard” effect on the patient’s condition. Moreover, the development of such hybrid measures is particularly relevant in rare diseases, considering that in addition to clinical scales, it makes sense to develop biomarkers for treatment concept evaluation or as surrogates [103]. A powerful related field is also emerging: the RWE. Actual patient registries, electronic health records, and wearable health monitoring devices provide large volumes of unconventional data. Their analysis allows testing of biomarker behavior in real-world settings, outside controlled trials, thereby supporting surrogate validity. Moreover, safety biomarkers (toxicology), pharmacokinetic data, and patient-reported outcomes are also increasingly integrated into multidisciplinary therapy evaluation. Thereby, instead of relying on a single marker, future practice tends toward synergy: clinical scale + biochemical surrogate + RWE. This comprehensive approach provides a more complete picture of a drug’s action and reduces the risk of misinterpretation.

### 8.4. Multi-Omics Biomarkers

One of the promising areas is the creation of composite “super-markers” that reflect the disease at different levels. The concept of multi-omic biomarkers involves combining several -omic layers (genome, transcriptome, proteome, metabolome, etc.) together with clinical information and even imaging data. Such a composite index can capture detailed pathology signature and be more accurate than traditional indicators. For instance, it was recently shown that multi-omics composite biomarker panels can detect more subtle changes in non-alcoholic steatohepatitis, with this combined “panel” marker outperforming individual markers in reflecting the complexity of disease pathogenesis and progression [113]. Obviously, such approaches require advanced computational methods and the collection of large datasets, but they enable capturing the complex nature of rare diseases. In the future, diagnosis and progression may be assessed not by a single biomarker, but by complex composites integrating patient’s genetics, metabolite profiles, gene expression, and more. Such complex models (e.g., using machine learning) already enable earlier and more precise patient stratification and treatment outcome prediction, reducing reliance on invasive procedures (e.g., biopsies) and accelerating therapy development.

## 9. Conclusions

Surrogate biomarkers play a key role in the development and evaluation of gene therapies for orphan diseases, especially given limited patient populations and the challenges of long-term clinical trials. They enable acceleration of drug approval, reduction of research timelines and costs, and objectification of therapeutic outcomes. However, their use requires strict validation, including evidence of biological plausibility, reproducibility, predictive value, and regulatory acceptance [5,8].

Regulatory approaches to surrogate markers vary by country. For example, FDA and EMA allow their use in accelerated approval programs (Accelerated Approval, Conditional Marketing Authorisation), but require later confirmation of clinical efficacy in post-marketing studies [10,12]. In Russia, although no formalized procedures equivalent to Western ones, steps are also being taken to integrate surrogate markers into regulatory practice, particularly in the context of rare diseases.

Preclinical studies based on transgenic and knockout animal models demonstrate the high relevance of surrogate biomarkers, such as enzyme or metabolite levels, for assessing the efficacy of gene therapies [46,48]. However, extrapolating these results to humans requires caution due to potential interspecies differences [68].

Key examples, such as surrogate marker applications in MLD, MPS I, and hemophilia B, highlight their importance for monitoring therapeutic response [80,92]. Nevertheless, instances where biomarker improvement was not accompanied by a clinical effect emphasize the need for careful validation and combination of surrogate endpoints with clinical outcomes [93,94].

Future perspectives for surrogate biomarkers are linked to the adoption of new technologies such as multi-omics and AI, which enable identification of complex disease signatures [105,113]. Expanding regulatory support and data standardization further contribute to accelerating their validation and implementation into clinical practice [110].

In conclusion, surrogate biomarkers remain indispensable tools in gene therapy for rare diseases, but their application must be based on strict scientific and regulatory standards. A comprehensive approach, combining biochemical markers with clinical data and RWE, will foster further progress in this field and improve the quality of life for patients.

## Figures and Tables

**Table 1:** Comparative analysis of regulatory approaches to surrogate endpoints (based on regulatory documents and guidelines issued between 2008 and 2025).

Regulator	Validated Surrogate	Candidate Surrogate	Approach to Use	Accelerated/Conditional Programs	Examples
FDA (USA)	Endpoint proven by multiple datasets as a reliable predictor of clinical benefit.	A marker with a justified hypothesis but not fully confirmed. Used in AA.	Validated surrogates are accepted in traditional NDAs/BLAs; “reasonably likely” surrogates are used for AA, with post-marketing confirmation. Actively consults on new markers.	AA, Fast Track, Breakthrough, Priority Review; Qualification Program (DDT ).	Lecanemab (AD, amyloid reduction by PET); multiple oncology drugs (ORR, PFS).
EMA (EU)	Does not formally define the term, but requires evidence of the marker’s association with clinical benefit.	Analogous to “candidate”, considered through a qualification procedure.	Surrogates may be used in conditional approval if benefit–risk is favorable and early access is needed. Biomarkers undergo CHMP qualification opinions. Early Scientific Advice on endpoints is encouraged.	CMA, Accelerated Assessment, PRIME; biomarker-method qualification procedure.	CMA for rare/severe diseases: drugs for oncology or rare genetic diseases. Qualified marker: GFR-slope for CKD.
Ministry of Health/Roszdravnadzor (Russia)	No formal surrogate definitions in regulations; primary requirement is efficacy based on clinical outcomes.	No clear classification; markers are considered within clinical trials.	Preference for “hard” clinical outcomes (survival, clinical status, etc.). Surrogates may be used if sufficient evidence of association with efficacy exists, often relying on international practice. Work is ongoing on simplified registration procedures.	Currently, there is no accelerated program equivalent to FDA/EMA. Elements of accelerated registration introduced in emergencies or drug shortages, (draft norms for “accelerated” or “conditional” registration under development).	Proposals for fast-track registration of foreign medicines in case of market shortages. In exceptional cases: approval of vaccines/therapies based on immune response (surrogate).
PMDA (Japan)	Requires strict biological rationale and statistical association with the clinical outcome.	Allowed if there is a reasonable link to the true endpoint.	Requires inclusion of secondary endpoints, including true outcomes, and comparative data in the Japanese cohort. Conditional approval possible for severe diseases lacking alternatives.	Conditional approval with mandatory post-marketing verification. Re-examination in 6–8 years.	Tumor response rate in oncology.
NMPA (China)	Must have biological plausibility and statistical association with clinical benefit.	Allowed for conditional approval in life-threatening diseases.	Requires mandatory post-marketing confirmation. Relies on international standards (ICH, FDA, EMA).	Conditional approval with confirmatory studies required.	MRD levels in multiple myeloma; change in BMD in osteoporosis.
Health Canada	A validated surrogate must predict clinical benefit.	Non-validated surrogates require confirmatory studies.	Evaluates the totality of evidence. Recommends discussing surrogate use with the regulator prior to study initiation.	NOC/c conditional approval with obligations for further studies.	Viral load suppression in HIV; PFS in oncology; antibody titers after vaccination.

## Data Availability

No new data were created or analyzed in this study. Data sharing is not applicable to this article.

## Abbreviations

AA	Accelerated Approval (FDA program)
AAV	Adeno-Associated Virus
ABR	Annualized Bleeding Rate
AD	Alzheimer’s Disease
AI	Artificial Intelligence
AIDS	Acquired Immunodeficiency Syndrome
ARSA	Arylsulfatase A gene/enzyme
BMD	Bone Mineral Density
CDE	Center for Drug Evaluation (China)
CHMP	Committee for Medicinal Products for Human Use (EMA)
CKD	Chronic Kidney Disease
CMA	Conditional Marketing Authorisation (EMA)
CNS	Central Nervous System
CSF	Cerebrospinal Fluid
DDT	Drug Development Tools (FDA initiative)
EAEU	Eurasian Economic Union
EEC	Eurasian Economic Commission
EGFR	Epidermal Growth Factor Receptor
EMA	European Medicines Agency
ERT	Enzyme Replacement Therapy
FDA	Food and Drug Administration (USA)
FIX/F9	Coagulation Factor IX/its gene
GAG	Glycosaminoglycans
Gb3	Globotriaosylceramide
GBA1	β-glucocerebrosidase gene/enzyme
GCP	Good Clinical Practice
GFR-slope	rate of decline of Glomerular Filtration Rate
GLA	α-galactosidase A gene/enzyme
GlcCer	Glucocerebroside
GLP	Good Laboratory Practice
GM2	Ganglioside GM2
GMFM	Gross Motor Function Measure
GTMP	Gene Therapy Medicinal Product
HbA1c	Glycated Hemoglobin
HDL-C	High-Density Lipoprotein Cholesterol
HEX	β-hexosaminidase
HEXA	β-hexosaminidase A subunit gene/enzyme
HEXB	β-hexosaminidase B subunit gene/enzyme
HIV	Human Immunodeficiency Virus
HPFB	Health Products and Food Branch (Canada)
HSCs	Hematopoietic Stem Cells
ICH	The International Council for Harmonisation of Technical Requirements for Pharmaceuticals for Human Use
IDUA	α-L-iduronidase gene/enzyme
LDL-C	Low-Density Lipoprotein Cholesterol
LSD	Lysosomal Storage Disorder
LTFU	Long-Term Follow-up
lyso-Gb1	Glucosylsphingosine (Gaucher disease biomarker)
lyso-Gb3	Deacylated Gb3 (Fabry disease biomarker)
lyso-GM2	Deacylated GM2 (Tay-Sachs disease biomarker)
MHLW	Ministry of Health, Labour and Welfare (Japan)
MLD	Metachromatic Leukodystrophy
MPS I	Mucopolysaccharidosis Type I
MRD	Minimal Residual Disease
MRI	Magnetic Resonance Imaging
NAA	N-acetylaspartate
NDA/BLA	New Drug Application/Biologics License Application
NMPA	National Medical Products Administration (China)
NOC/c	Notice of Compliance with Conditions (Canada)
ORR	Objective Response Rate
OS	Overall Survival
PET	Positron Emission Tomography
PFS	Progression-Free Survival
PMD Act	Pharmaceuticals and Medical Device Act (Japan)
PMDA	Pharmaceuticals and Medical Devices Agency (Japan)
PNS	Peripheral Nervous System
PRIME	Priority Medicines (EMA program)
PSA	Prostate-Specific Antigen
RCT	Randomized Controlled Trial
RECIST	Response Evaluation Criteria in Solid Tumors
RWD	Real-World Data
RWE	Real-World Evidence
TSD	Tay–Sachs Disease
